# Inflammation, Autoimmunity, and Infection in Fibromyalgia: A Narrative Review

**DOI:** 10.3390/ijms25115922

**Published:** 2024-05-29

**Authors:** Marino Paroli, Chiara Gioia, Daniele Accapezzato, Rosalba Caccavale

**Affiliations:** Department of Clinical, Internal, Anesthesiologic and Cardiovascular Sciences, Sapienza University di Roma, 00185 Rome, Italy; chiara.gioia@uniroma1.it (C.G.); daniele.accapezzato@uniroma1.it (D.A.); rosalba_caccavale@yahoo.it (R.C.)

**Keywords:** fibromyalgia, immune system, autoimmunity, inflammation, infection

## Abstract

Fibromyalgia (FM) is a chronic disease characterized by widespread musculoskeletal pain of unknown etiology. The condition is commonly associated with other symptoms, including fatigue, sleep disturbances, cognitive impairment, and depression. For this reason, FM is also referred to as FM syndrome. The nature of the pain is defined as nociplastic according to the latest international classification and is characterized by altered nervous sensitization both centrally and peripherally. Psychosocial conditions have traditionally been considered critical in the genesis of FM. However, recent studies in animal models and humans have provided new evidence in favor of an inflammatory and/or autoimmune pathogenesis. In support of this hypothesis are epidemiological data of an increased female prevalence, similar to that of autoimmune diseases, and the frequent association with immune-mediated inflammatory disorders. In addition, the observation of an increased incidence of this condition during long COVID revived the hypothesis of an infectious pathogenesis. This narrative review will, therefore, discuss the evidence supporting the immune-mediated pathogenesis of FM in light of the most current data available in the literature.

## 1. Introduction

Fibromyalgia (FM) is a syndrome of unknown cause characterized by chronic widespread musculoskeletal pain that lasts for more than three months, often accompanied by symptoms such as fatigue, non-restorative sleep, cognitive impairment, short-term memory deficit, headache, irritable bowel, anxiety, and depression [1,2]. FM pain is considered nociceptive type pain (NcpIP) according to the most recent International Association for the Study of Pain (IASP) definition. NcpIP is currently defined as pain that results from altered nociception, despite the fact that there is no clear evidence of actual or threatened tissue damage causing peripheral nociceptor activation or evidence of disease or injury to the somatosensory system causing the pain. However, this new definition has been criticized by many authors for its perceived lack of clinical utility and the vagueness of the definition [3,4].

FM was officially recognized in 1990 when the American College of Rheumatology (ACR) first established diagnostic criteria based on the elicitation of pain at the appropriate pressure of 11 out of 18 specific body points or tender points. In 2010, these criteria were updated by introducing the concept of pain areas and FM syndrome. In 2016, additional criteria were proposed but not yet universally accepted for clinical diagnosis [5]. 

Fibromylagia has a significant impact on the patient’s quality of life. It is also responsible for a high rate of sick leave, which can generate financial problems for both patient and employer, considering its high prevalence, reaching more than 6% of the population in some studies [6,7,8]. Although the clinical features of FM have been satisfactorily elucidated, its etiology still remains a medical mystery. 

Since pain is the hallmark of the disease, most research has focused on the pathogenesis of its origin. Several studies have shown that in patients with FM, there is abnormal amplification of pain at the central nervous system (CNS) level, as also demonstrated by magnetic resonance imaging (MRI) studies of the brain. The origin of pain in FM has traditionally been attributed to emotional stress and psychosocial trauma in predisposed patients. However, this psychodynamic interpretation, at least in part, has recently been challenged [9]. Studies using spectroscopic magnetic resonance imaging (MRSI) have shown an abnormal thermal response after stimulation of immunity with endotoxin challenge in women with FM, suggesting a link between chronic widespread pain and the immune system [10]. In addition, a signature of genes encoding pro-inflammatory molecules produced by cells of the innate immune system, such as dendritic cells and neutrophils, has been identified in FM patients with associated depression [11]. It was also reported that in the peripheral blood of patients with FM, concentrations of pro-inflammatory cytokines, including interleukin (IL)-6, IL-8, IL-17, interferon (IFN)-γ, and tumor necrosis factor (TNF)-α, and various chemokines were associated with symptom severity [12,13,14,15,16,17,18]. 

In an interesting study, a Mediterranean diet enriched with tryptophan and magnesium was shown to reduce symptoms of anxiety, mood disorders, eating disorders, and body image dissatisfaction in patients with FM. This indirectly suggests that a diet containing instead foods with pro-inflammatory action may contribute to neuroinflammation and worsening of symptoms in these patients [19].

Of particular interest is the observation that FM is significantly associated with autoimmune/inflammatory conditions, such that it can be considered a co-morbidity of diseases of immunologic origin [20,21,22]. Data from a large meta-analysis study showed that about 21% of patients with rheumatoid arthritis (RA), 13% with axial spondyloarthritis (axSpA), and 18% with psoriatic arthritis (PsA) also have FM [23]. The concomitance of FM and axSpA even initially prevented FDA approval of anti-TNF-alpha (TNFi) biologics for patients with the non-radiographic form of the disease (nr-axSpA) because FM could have significantly participated in low back pain despite the presence of obvious signs of active sacroiliitis evidenced by MRI or the presence of the axSpA-associated human leukocyte antigen (HLA)-B27 allele. A subsequent study showed that patients with high axSpA disease activity have concomitantly higher severity of associated FM [24,25,26]. 

The purpose of this narrative review is to provide an overview of the latest evidence in favor of an immune-mediated pathogenesis of FM, highlighting and discussing sometimes overlooked aspects of this disease that support the hypothesis of the autoimmune/inflammatory nature of FM.

## 2. The Innate Immune System

### 2.1. The Role of Mast Cells

The role of mast cells has been explored in the inflammatory genesis of FM syndrome. Indeed, inhibition of these cells has been correlated with decreased pain in experimental models [27,28]. In addition, these cells are able to secrete several interleukins involved in central nervous system (CNS) inflammation, such as IL-17, IL-6, and tissue growth factor-β (TGF-β) [29]. Mast cells can also produce IL-1 after stimulation of high-affinity IgE receptors (FcεRI) or toll-like receptors (TLRs) [30]. In this regard, IL-1 has been shown to play a key role in FM syndrome and has been identified in the skin of these patients [31,32]. Proteolytic activation of IL-1 is controlled by inflammasomes, which are large multiprotein signaling platforms. This suggests that targeting the inflammasome could be a potential therapy for IL-1-mediated pain syndromes, including FM [33,34]. It has also been shown that IL-1 production is induced by mast cells following pathogenic infection, possibly associated with pain syndrome [35,36]. 

Since bacterial and viral infections have been implicated in the induction of FM [37], mast cells could mediate the induction of FM through an inflammatory response following a triggering event of an infectious nature. It was also reported that the number of mast cells in skin biopsies from patients with FM was found to increase approximately threefold compared with healthy subjects [38,39]. Mast cells have also been shown to disrupt the blood-brain barrier, allowing various proinflammatory substances to enter brain tissue [40]. It is noteworthy that the pain mediator Substance P (SP), elevated in patients with FM syndrome [41], is able to stimulate mast cells, demonstrating that stimulation between CNS and mast cells is bidirectional [42]. In addition, the interaction of mast cells with microglia can cause their activation to be mediated by the production of pro-inflammatory substances such as histamine and tryptase [43]. Microglia cells can, in turn, secrete proinflammatory cytokines within the brain, particularly at the thalamic level, where they can induce chronic pain [44]. All this evidence suggests that mast cells play a key role in pain syndromes, including FM [45].

### 2.2. The Role of Neutrophils

Neutrophils are key cells of innate immunity [46,47]. Although some studies have suggested that these cells might have an inhibitory action on pain through the expression on their surface of opioid receptors and the ability to synthesize anti-inflammatory substances [48,49], other studies, however, have emphasized their possible role in the amplification of nociceptive pain [50,51]. 

Some authors have reported high levels of neutrophils in the peripheral blood of patients with FM syndrome. In these patients, neutrophils have demonstrated high chemotactic and microbial killing capacity [52,53]. In addition, many cytokines identified in patients’ serum with FM, such as IL-6, IL-8, and TNF-α, are produced by neutrophils [54,55]. In this regard, it was reported in one study that inhibition of IL-6 activity by the monoclonal antibody tocilizumab was able to reduce pain in patients with FM [56]. Although neutrophils are not normally present in the central nervous system, they can reach this anatomical site under pathological conditions, as demonstrated in animal models [57,58]. 

In a recent study, in an animal model of artificially induced diffuse pain syndrome clinically similar to FM, it was reported that transfer of cells from these mice to naïve recipient mice induced a pain syndrome similar to that of the source mice. The depletion of neutrophils from the transferred cells was not accompanied by the induction of pain, demonstrating the central role of these cells in the genesis of the pain syndrome. In addition, neutrophil infiltration was found in the sensory ganglia of experimental mice. In the same study, neutrophils obtained from FM patients but not from controls and transferred into mice similarly induced diffuse pain syndrome [59,60]. All this evidence suggests that neutrophils may be a potential target in the treatment of FM.

### 2.3. The Role of Microglia System

Microglia cells are phagocytic cells resident in the central nervous system, and their main function is to defend brain tissue from attack by pathogenic microorganisms [61,62]. These cells are considered the macrophages of the CNS and, as such, can switch from a pro-inflammatory M1-type phenotype, which releases TNF-α, IL-1-β, and IL-6, to an anti-inflammatory M2-type phenotype, which produces IL-10, IL-4, and IL 13. The transition from M1 to M2 and vice versa is strictly dependent on the neuronal microenvironment [63,64]. M1 microglia induce both nociceptive and nociplastic pain, while M2 microglia exert an inhibitory effect on pain [65,66]. 

It has recently been reported that naltrexone, an opioid receptor antagonist, can inhibit the pro-inflammatory action of microglia by modulating M1/M2 switching through stimulation of toll-like TLR-4, thereby reducing chronic pain resulting from neuroinflammation. This finding has possible important implications for FM patients [67]. Recently, it has been shown that in patients with FM, there is an imbalance in the M1/M2 ratio in favor of M1 cells, while the levels of M2 microglia markers have decreased [68]. 

Animal models and human studies have also shown that activation of microglia cells is a major contributor to the chronic widespread pain characteristic of FM [69,70,71,72]. Microglia cells also expressed high levels of substance P (SP), an important mediator of nociplastic pain in FM [73]. Such evidence suggested the possibility of pharmacologically modulating the M1/M2 ratio of microglia cells to reduce pain of neuroinflammatory origin. To this end, naloxone [74], IL-5 [75], infliximab [76], and dextromethorphan [77], through different mechanisms of action, have been used to suppress pain with relative success.

### 2.4. The Role of Natural Killer Cells

Natural Killer (NK) cells constitute a population of the innate immune system derived from a lymphoid precursor common to B and T lymphocytes. Although NK cells do not express the T-cell receptor (TCR) and the CD3 molecule, they are activated or inhibited by specific major histocompatibility complex (MHC) receptors and are believed to be a transitional cell subset between innate and adaptive immunity [78]. 

NK cells are able to respond rapidly against virus-infected cells or cancer cells [79,80]. NK cells express Mu opioid receptors on their surface [81,82]. It has been reported that opioids may have an inhibitory effect on NK cell proliferation, as the number of such cells in the peripheral blood of patients treated with exogenous opioids was found to be reduced compared with untreated subjects [83]. In addition, several studies have shown that the cytotoxic activity of these cells is inhibited by opioids in both animal models and humans [84,85]. However, it has been observed that the immunomodulating effect on NK cells depends on the type of opioid considered [86]. 

In an interesting study, increased expression of NK cell activation ligands was found in skin biopsies of FM patients at the subepidermal nerve level and the presence of NK cells near peripheral nerves, suggesting that these cells play an important role in pain induction [87]. In addition, increased expression of NK cell activation ligands was found in skin biopsies of FM patients at the subepidermal nerve level, and NK cells were found near peripheral nerves [88]. In another large study, the key role of NK cells, particularly with CD56^bri^ phenotype, in mediating pain in patients with FM was recently reported [88]. 

Although it is unclear from all this evidence whether NK cells play a functional role in pain modulation in FM, it has been suggested that the number of NK cells expressing opioid receptors in peripheral blood could be a biomarker for FM diagnosis and disease activity.

## 3. The Adaptative Immune Response

### 3.1. The Role of T Cells

A possible role of T cells in the pathogenesis of FM was initially suggested by the observation in transgenic animal models that, after stimulation of nociceptive receptors, afferent neurons in the peripheral nervous system were able to secrete IL-17 mediated by T cells expressing both α/β- and γ/δ-type TCR [89]. Some evidence shows that there is a cross-talk between T cells and the central and peripheral nervous system. For example, glutamate can induce T-cell activation, while dopamine and other neurotransmitters can influence T-cell differentiation [90]. 

It has also been shown in mouse models that after T-cell depletion, some nerve pain responses are abolished, including central tactile sensitization after sciatic nerve ligation [91,92]. It is also interesting to note that in experimental models, hypersensitivity to pain after mechanical trauma was associated with an increase in circulating CD4^+^ and CD8^+^ T cells. The higher prevalence of this phenomenon in women suggests a gender difference in pain-cell interaction. This is in agreement with the higher prevalence of FM in females compared with males [93]. 

In humans, previous studies conducted on the relationship between FM and T cells showed that T lymphocytes isolated from the peripheral blood of FM patients were anergic compared with control subjects, as evidenced by lower IL-2 production under the same experimental conditions [94]. Subsequently, it has been reported that T lymphocytes from patients with FM syndrome show reduced expression of activation markers on their surface [95]. However, this result was not confirmed by other studies that showed an increase in the number of activated CD25^+^ T lymphocytes in FM patients compared with healthy subjects [96]. 

The studies described so far do not definitively clarify whether alterations in T-cell populations are the cause or effect of the pain stimulus in these subjects. For example, one study found a reduction in the number of cytotoxic-acting CD8^+^ T cells in patients with chronic pain, but it could not prove whether this event preceded or followed the onset of the pain syndrome [97]. 

However, another interesting observation is that in FM patients carrying mutations in the promoter region of the gene encoding for the serotonin transporter 5-HTTLPR, an increase in the number of activated T cells was observed. This suggested a correlation between the adaptive immune response and serotonin, a neuronal mediator involved in the stimulation of nociceptive sensory nerves [98]. The role of T cells in FM is also suggested by the increased levels of chemokines in patients’ blood. Indeed, these substances facilitate the recruitment of T cells through chemotaxis [15,54]. 

An extensive study of the phenotype of circulating T cells and their cytokine profile has shown that T cells in FM subjects predominantly belong to the T helper (Th)-1 subgroup. This subpopulation is characterized by the production of pro-inflammatory cytokines, including, in particular, TNF-α and IFN-γ. FM patients treated with hyperbaric therapy for pain relief revealed a reduction in both circulating Th1 cells and serotonin, further suggesting the involvement of the neuro-immunological axis in the widespread pain symptoms of these patients [99]. 

The possible involvement of Th17 cells was also reported, as transcriptome analysis revealed an IFN signature from cells in the serum of FM patients [100]. Studies of cannabinoids used in the treatment of pain in patients with FM syndrome have been shown to modulate cytokine production by T lymphocytes, thus suggesting that their analgesic effect is at least in part mediated by adaptive immunity, specifically through inhibition of pro-inflammatory cytokine production and stimulation of those with anti-inflammatory activity [101,102].

Although, as indicated above in this review, a causal relationship between alterations in CD4^+^ T-cell activity and the pathogenesis of FM cannot be established with certainty on the basis of all reported findings, the hypothesis that this widespread pain syndrome may have an autoimmune genesis mediated by autoreactive T cells is highly suggestive [103]. The observation of the possible involvement of pathogens, vaccine adjuvants, and the higher prevalence in the female sex have pointed out further clues in favor of this hypothesis [104,105,106,107]. Some studies have also associated increased prevalence in FM of HLA class II alleles involved in autoimmunity [108]. It should also be emphasized that although environmental conditions, particularly stress, can influence the genesis of autoimmune processes [109], animal models have recently shown that an aberrant cellular T response can, in turn, induce stress conditions [110,111]. 

Moreover, in animal models of anxiety, CD4^+^ lymphocyte depletion prevents stress-related behaviors, just as adoptively transferred CD4^+^ T cells induce the stress phenotype in healthy recipient mice [112,113]. Immunodeficient mice also appear to be protected from laboratory-induced stress [114,115,116]. Other studies have demonstrated alterations in genes encoding mitochondrial proteins in T cells of stressed mice, with consequent altered morphology [117]. In more detail, mitochondria were found to divide by segregating into two separate mitochondrial organelles, a phenomenon termed mitochondrial fission. This phenomenon, in turn, induces the development of Th1 cells and the inhibition of regulatory T cells (Tregs) with anti-inflammatory action through the accumulation of interferon regulatory factor-1 (IRF-1) [118]. 

Finally, Treg has been shown to modulate IL-5 production by Th2 cells and, consequently, the analgesic activity induced by this cytokine [119]. The interaction between stress, altered mitochondrial T-cell RNA, and FM certainly needs further investigation for its potential pathogenic implications. 

### 3.2. The Role of B Cells and Autoantibodies

B cells play a key role in autoimmune processes. Their function is multifaceted and consists of the production of autoantibodies, the presentation of self-antigens to T lymphocytes, and the ability to secrete cytokines with proinflammatory activity. However, the role of B cells in the possible autoimmune pathogenesis of FM has not yet been thoroughly studied. In a recent study, an increase in B cells expressing mu-opioid receptors was reported in patients with FM [120]. 

In another study, a comprehensive analysis of the B-cell transcriptome in FM patients was conducted. The results showed overexpression of many IFN-regulated genes, indicating that this IFN signature may be caused by the interaction of IFN-secreting cells of the innate and adaptive immune system with B cells and that this may play a key role in the pathogenesis of FM. This suggests, among other things, that a therapeutic approach to counteract dysregulated interferon production could benefit FM patients [121].

**Figure 1:**
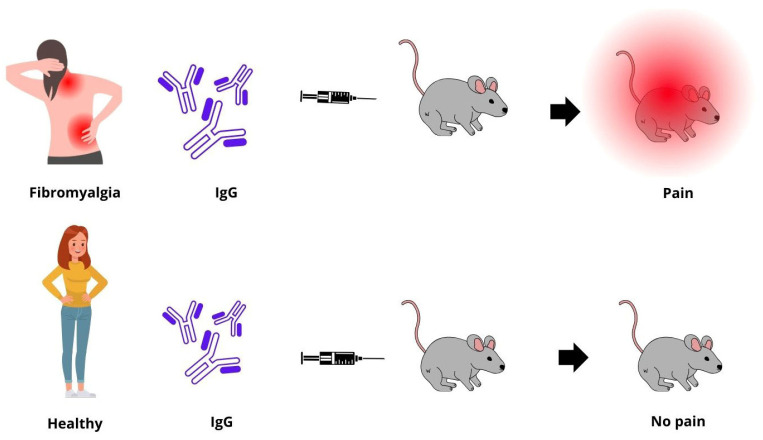
Passive transfer of serum IgG from fibromyalgia patients into mice induces fibromyalgia-like pain. Goebel et al. [122] recently demonstrated in an elegant experimental model that passive transfer of IgG-class immunoglobulin from fibromyalgia patients to mice induced sensory hypersensitivity through sensitization of nociceptive neurons. This experiment represented a breakthrough in understanding the pathogenesis of fibromyalgia, strongly suggesting an autoimmune mechanism mediated by antibodies against satellite glial cells and neurons.

Interest in the role of autoantibodies in the pathogenesis of FM received a definite boost after the recent publication of a study conducted in an animal model showing that passive transfer of IgG-class antibodies obtained from the serum of FM patients, but not from that of controls, is able to induce a state of hypersensitivity to pain by sensitizing the afferent nociceptive neurons of injected mice [122]. Figure 1 summarizes the experimental design. In the same study, these antibodies were shown to bind the surface of satellite glial cells, neurons, myelin fibers, macrophages, and endothelial cells found in the dorsal root ganglia. The same antibodies were also detected in the spinal ganglia of FM patients. In a later study, it was shown that the typical symptoms of FM were particularly pronounced in subjects whose titer of anti-spinal ganglion autoantibodies was very high [123,124]. These antibodies, however, have not been found in all FM patients, suggesting that they could participate in the pathogenesis of FM only in a subgroup of subjects. In any case, the pathogenetic role of autoantibodies in experimental models and in humans that emerged from the above studies greatly strengthened the hypothesis of autoimmune pathogenesis of FM. It is also conceivable that other autoantibodies responsible for widespread pain syndromes have not been identified so far [125,126].

## 4. The Role of Pathogens

Pathogen infections have long been and still are strongly implicated in the pathogenesis of FM by many authors [127]. Studies on hepatitis C virus (HCV) infection have found an increase in the prevalence of not only chronic pain but also other symptoms associated with FM, such as fatigue and depression [128,129,130]. 

The presence of high FM in human immunodeficiency virus (HIV)-infected patients has also been widely described [131,132]. In Lyme disease, a bacterial infection caused by Borrelia burgdorferi, FM has been described as a frequent co-morbidity. Interestingly, antibiotic therapy fails to regress the pain syndrome, suggesting the initiation of a chronic autoimmune process [133,134]. Epstein-Barr virus (EBV) [135], gut bacteria [136], and *Helicobacter pylori* [137] are other pathogens that have been associated with FM. A recent boost to the study of correlations between pathogens and FM has come from the COVID-19 pandemic. 

In a recent study using COVID-19 and FM blood transcriptome data and machine learning studies, a number of FM-related genes were identified that were activated after SARS-CoV-2 infection and that were particularly manifested during “long-COVID” co-infection. These genes are related to the synthesis and regulation of various cytokines. It was, therefore, hypothesized that these cytokines are a key mediator of pain in patients with FM. Other genes identified in the study and related to post-COVID FM were related to leukotriene synthesis, activation of innate immunity, and oxidoreductive stress [138,139]. 

Thus, the symptoms of long COVID include several typical manifestations of FM, such as cognitive impairment, fatigue, musculoskeletal pain, depression, anxiety, and sleep disturbances [140,141,142]. For these reasons, the definition of post-COVID FM has been coined [143]. It is possible that CNS hyperexcitability, which occurs particularly during the cytokine storm that characterizes severe COVID-19, indicates the possibility of an inflammatory origin of FM following severe SARS-CoV-2 infection [144]. 

However, a possible pathogenetic link with the antiviral immune response has been hypothesized even in mild cases of COVID-19 [145]. Among various pathogenic causes, some studies have investigated mitochondrial dysfunction common to patients with COVID-19 and FM. This dysfunction leads to abnormal production of reactive oxygen species (ROS) with possible induction of chronic pain [146,147]. In summary, it can be speculated that the close relationship between long COVID and FM may be an important clue to clarify aspects of FM [148]. 

**Figure 2:**
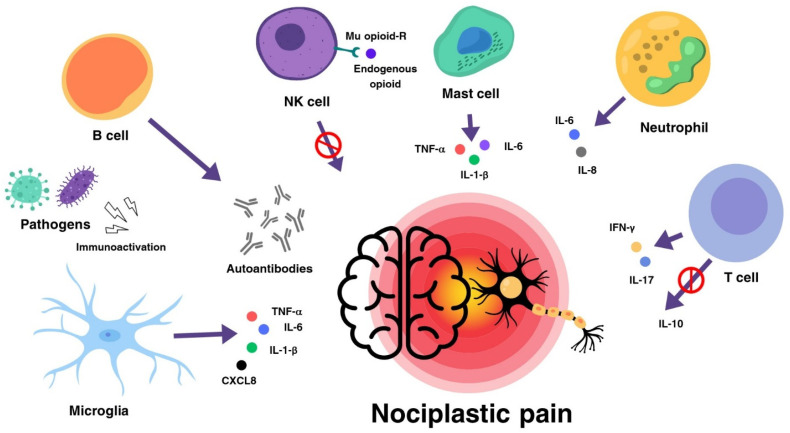
Induction of nociplastic pain by cells of the immune system. Cells of the innate and adaptive immune systems may participate in the genesis of nociplastic pain in FM. Microglia cells, neutrophils, and mast cells produce interleukins and chemokines. B cells produce autoantibodies against nerve cells. T cells produce both inflammatory cytokines, such as IFN-γ and IL-17, and anti-inflammatory cytokines, such as IL-10. NK cells are believed to inhibit pain through their stimulation of Mu-type opioid receptors. Viral and bacterial pathogens can act as stimulators of the immune system. The red circle banned sign above the arrow indicates the inhibitory action by the cell.

The inflammatory hypothesis suggests the possibility of evaluating immunosuppressive therapeutic possibilities for FM, given the common immunologic aspects between this disease and COVID-19, such as drugs used for the COVID-19, such as tocilizumab or Janus kinase inhibitors (JAKis) [149]. The role of innate and adaptive immune cells in the induction of nociplastic pain in FM is summarized in Figure 2.

## 5. Anti-Inflammatory Activity of Current Therapies in FM Syndrome

Duloxetine and milnacipran, both selective serotonin and norepinephrine reuptake inhibitors (SSNRIs), and the antiepileptic pregabalin are the only drugs approved for the treatment of FM by the Federal Drug Administration (FDA) in the United States. Their use takes advantage of their pain-inhibiting action on the central nervous system. 

However, there is growing interest in the anti-inflammatory activity of these drugs in light of the hypothesis of an autoimmune inflammatory pathogenesis of FM. In a recent study, the authors used neutrophil-to-lymphocyte ratio (NLR), platelet-to-lymphocyte ratio (PLR), and mean platelet volume (MPV) as surrogate markers of inflammation [53,150]. In a prospective observational study, it was reported that the NLR was significantly higher in patients with FM than in controls. Treatment with duloxetine led to a reduction in this ratio, indicating that this antidepressant medication may be effective on pain in these patients by reducing the inflammatory state [151]. 

Regarding the anti-inflammatory activity of the antiepileptic pregabalin, a study was conducted in female patients with FM syndrome. In that study, it was reported that pregabalin was associated with a decrease in serum of several cytokines such as IL-6, IL-17, TNF-α, and IFN-γ in treated patients [152]. Although these studies do not demonstrate that the efficacy of SSRIs and antiepileptic drugs is related solely to anti-inflammatory effects, they provide an interesting indirect suggestion in favor of the inflammatory pathogenesis of FM syndrome. 

Among nonpharmacological therapies, exercise-based interventions (EBI) are often recommended for patients with FM syndrome. A systematic search of several electronic databases demonstrated, through a meta-analysis, a significant reduction in ESR and pro-inflammatory interleukin IL-8 levels in patients with FM syndrome who practiced EBI. Although the authors themselves concluded that their work should be interpreted with caution, it represents a further suggestion to the inflammatory hypothesis in the pathogenesis of FM syndrome [153]. 

Another nonpharmacological therapy sometimes used to treat FM syndrome is mindfulness-based stress reduction (MBSR). In a randomized trial involving only female patients, this treatment approach was shown to prevent the reduction of anti-inflammatory interleukin IL-10. In addition, high levels of pro-inflammatory substances before therapy, including CX-10 chemokines, were greatly reduced. In addition, high levels of pro-inflammatory substances before therapy, including chemokines CXCL8 and IL-6, impaired the effectiveness of this therapy [154].

## 6. Discussion and Conclusions

**Figure 3:**
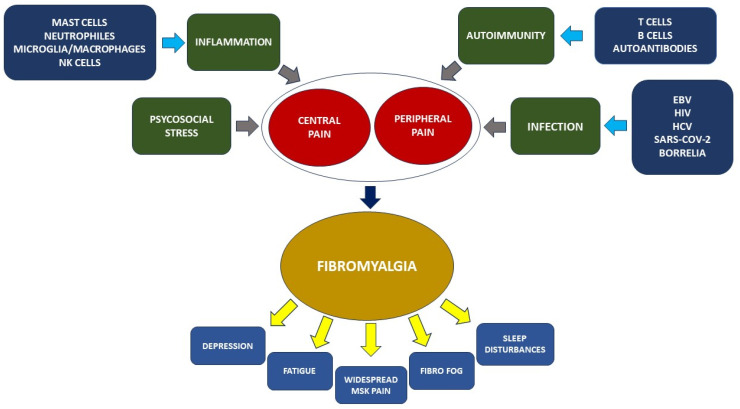
Factors participating in the pathogenesis of fibromyalgia. Several factors are involved in the genesis of fibromyalgia (FM). Traditionally, psychosocial stress has been considered the main event in individuals predisposed to the activation of both central and peripheral pain sensitization. However, recent findings have demonstrated the key role played by the immune system. Inflammation mediated by mast cells, neutrophils, microglia cells, and natural killer (NK) cells produces several proinflammatory cytokines and chemokines that contribute to neuroinflammation and the subsequent increase in pain sensitization. On the other hand, recent studies have also involved adaptive immunity, demonstrating the role of T cells, particularly T helper (Th)-1 and Th17 cells capable of producing pro-inflammatory cytokines, and B cells through the production of neuron-specific autoantibodies, as demonstrated through animal models of passive IgG transfer in experimental animals. Finally, infections play an important role. In particular, infection with SARS-CoV-2, the causative agent of COVID-19, is believed to be responsible, through still unknown mechanisms, for the increased incidence of FM reported during the so-called “long COVID”. Once grafted, fibromyalgia has a chronic course and, in addition to widespread musculoskeletal pain, is accompanied by various debilitating symptoms such as depression, fatigue, cognitive impairment, also referred to as fibro fog, and sleep disturbances.

The most recent studies have shown that FM is a condition characterized by a complex pathogenesis. The hypothesized mechanisms in the pathogenesis of FM are schematically showed in Figure 3. Theories that until recently considered this condition as an effect predominantly associated with psychophysical stress or trauma of psychological origin no longer seem able to adequately explain the onset of this pathological condition characterized by chronic widespread pain.

Several pieces of evidence point out that cells of both the innate and adaptive immune systems may contribute decisively to the pathogenesis of FM. Mast cells, with their ability to cross the blood-brain barrier and activate microglia cells, may play a very relevant role in neuroinflammation. Microglia cells, being able to switch from a pro-inflammatory M1 phenotype to an anti-inflammatory M2 phenotype, may represent an interesting therapeutic target, possibly able to modulate the balance of the M1/M2 phenotype. NK cells also appear to play both pro-inflammatory and anti-inflammatory roles. Opioid receptors present on the membrane of these cells may explain at least in part the pain-relieving effects of this class of drugs in FM. 

As for the cells of the adaptive system, Th1- and Th17-type helper T cells seem to play an important role. These cells, in addition to producing pro-inflammatory soluble substances, have been shown to interact with pain-related neuronal mediators, including serotonin. This may explain the effects of some antidepressant drugs in the treatment of FM. 

More recently, the role of B lymphocytes has been emphasized, both in their function of antigen presentation to T lymphocytes and in the production of cytokines and antibodies. In the latter regard, recent studies in animal models have shown that passive transfer of IgG from patients with fibromyalgia can induce an FM-like pain syndrome in experimental mice. The nature of these antibodies has not yet been defined, but these findings strongly suggest an autoimmune component of FM. It appears increasingly clear from an experimental and more organicist approach that factors such as genetic predisposition associated with environmental factors come into play. Infections probably play a key role in the onset of FM, but the downstream mechanisms responsible for the persistence of chronic pain always seem to be mediated by autoimmune and inflammatory phenomena.

In conclusion, the data reported in this review support the autoimmune and inflammatory pathogenesis of FM. This suggests that a better understanding of these immune-mediated mechanisms could be exploited to develop innovative therapies for this, in many ways, mysterious disease. It must be emphasized, however, that this review specifically examined the autoimmune, inflammatory, and infectious aspects involved in the genesis of FM. Therefore, it does not claim to be a comprehensive review of this disease, as it did not consider experimental studies that examined other factors and provided different interpretations of the pathogenesis of the disease, including a vegetarian diet and manual therapy [155,156].

Further studies in all these regards are warranted to better elucidate the pathogenesis of a very complex condition, also in order to allow increasingly accurate diagnosis of FM and to set up possible targeted and effective treatments, all of which are unmet needs of this highly disabling condition that has a great impact on the quality of life of those affected.

## Data Availability

All data generated and analyzed in this review were not included.
